# Health insurance and maternal, newborn services utilisation and under-five mortality

**DOI:** 10.1186/s13690-015-0101-0

**Published:** 2015-12-29

**Authors:** Samuel Bosomprah, Peter Luigi Ragno, Clemens Gros, Hari Banskota

**Affiliations:** Department of Biostatistics, School of Public Health, University of Ghana, Legon, Accra, Ghana; Health and Nutrition Department, UNICEF Ghana Office, Accra, Ghana; Health and Nutrition Department, University of Manchester, Accra, Ghana

**Keywords:** Health insurance, Maternal, Newborn, and Child health

## Abstract

**Background:**

Ghana’s National Health Insurance Scheme (NHIS) was introduced in 2005 as a demand side intervention to remove financial barriers to accessing health services. After almost a decade of implementation, this study aims to investigate the association of NHIS membership with antenatal visits (ANC), postnatal visits (PNC) and under-five mortality, using data from the most recent Multiple Indicator Cluster Survey (MICS).

**Methods:**

The survey was nationally representative and used a two-stage sample design to produce separate estimates for key indicators for each of the ten regions in Ghana. A generalised linear model (GLM) with binomial-family logit-link was used to estimate the effect of NHIS membership on each of the MNCH service utilisation indicators, adjusting for relevant confounding factors. Using birth history data, the Cox proportional hazard regression model was used to estimate the effect of NHIS membership on the incidence of under-five deaths, adjusted for wealth quintiles and other potential confounders.

**Results:**

The results support the role of health insurance membership in improving access to maternal and child health services, including antenatal care (ANC4+ adjusted OR = 1.94; 95 % CI = [1.28, 2.95]; *P* < 0.01), and content of antenatal care (adjusted OR = 2.05; 95 % CI = (1.46, 2.90); *P* < 0.0001). However, the study failed to show evidence of association of NHIS membership and under-five mortality (adjusted hazard rate = 0.86; 95 % CI = [0.64, 1.14]; *P* = 0.30).

**Conclusions:**

National health insurance membership is associated with increased access to and utilisation of health care but not with under-five mortality.

## Background

Strong health systems improve the health status of the whole population, especially the poor among whom ill health and poor access to health care tends to be concentrated [1]. It is known that increasing women’s access to skilled delivery care, and children’s access to evidence based health interventions effectively protect the mother and child from illness and death. Conscious of this evidence, the Ghana Ministry of Health adopted several strategies to facilitate access to health services [2, 3]. On the supply side, a UNICEF-funded High Impact Rapid Delivery (HIRD) approach to deliver maternal and child health services to ensure universal coverage of a package of maternal and child health interventions was a key strategy. In an effort to address supply side constraints, basic health services were moved as close as possible to the communities through the Community Health and Planning Services (CHPS), a ‘close to client’ model of service delivery. On the demand side, following the evidence that user fees discourage the poor from accessing and using health services [4–6]; the Government of Ghana launched a National Health Insurance Scheme (NHIS) in 2005.

Global evidence suggests that health insurance increase health service access and utilisation [7]. Owoo et al. [8] found that women who sign up to health insurance were more likely to use antenatal services compared with women who do not. In an evaluation of the Ghana National Health Insurance Scheme, Mensah et al. [9] concluded that women who are enrolled are more likely to give birth in hospitals and have skilled attendant at birth. They are also more likely to have fewer birth complications and experience fewer infant deaths. Brugiavini and Pace [10] also found that NHIS membership positively affects the probability of institutional delivery and assistance by skilled personnel during delivery. Sulzbach [11] compared data in two districts in Ghana before (in 2004) and after NHIS implementation (in 2007) and found evidence of an increase in access to formal care among NHIS members as well as a significant decrease in health care expenditure. However, they found no evidence of a difference in maternal care between women who enrolled in NHIS and those who did not.

Health insurance has also been linked to improved maternal, newborn and child health outcomes. For example, the Medicaid program has been shown to be associated with reduction in infant mortality and low birth weight in the United States of America (USA) [12, 13]. Similarly, the State Children’s Health Insurance Program (SCHIP) has been shown to improve child health outcomes in the USA [14, 15]. In Taiwan, the nationwide health insurance program has been linked to reduced incidence of death among young children [16]. A study conducted in Colombia showed that health insurance can free financial resources for households [17]. In Ghana, the authors have found only one study [18] that showed evidence of association between national health insurance and anaemia in children and a randomized controlled trial in which Ansah et al. [19] concluded that removing out-of-pocket payments improved the health care-seeking behavior of mothers but did not improve child health outcomes. But none of these two studies examined the role of NHIS membership on under-five mortality. Therefore, this article aims to investigate the association of NHIS membership with antenatal visits (ANC), postnatal visits (PNC) and under-five mortality using the data from the Multiple Indicator Cluster Survey (MICS). By doing so the paper examines if the membership to the NHIS can contribute on one hand, to increasing access and utilization of health services, while on the other, to an improved health outcome for children.

## Methods

### Ethical statement

Ethical approval for the Multiple Indicator Cluster Survey (MICS) was obtained from the Ghana Health Service. The data available for this study cannot be linked to an individual who participated in the study.

### Data source

The MICS 2011 was used for this analysis [20]. The choice of using MICS 2011 data was made to allow for a sufficient time period after the introduction of NHIS, yielding a greater likelihood of observing its potential effects on maternal and child health service utilisation and outcomes. The survey is nationally representative and used a two-stage sample design to produce separate estimates for key indicators for each of the ten regions in Ghana. The first stage involved systematically selecting clusters (called enumeration areas or EAs) with probability proportional to size from an updated master sampling frame constructed from the Ghana Population and Housing Census (2010) [21]. The second stage of selection involved the systematic sampling of the households listed in each cluster. The MICS (2011) duly interviewed 11,925 households. In these households, 10,627 women aged 15–49 years were duly interviewed giving a response rate of 97 percent. Complete responses were obtained on 7550 children under age 5 from their mother/caregiver. The number of women age 15–49 who had a live birth in the two years preceding the survey was 2,528. Further details of the sample design and questionnaire are described elsewhere [20].

### Statistical analysis

In this analysis, the main exposure of interest was NHIS membership defined as having a valid insurance card, which was seen and confirmed by the interviewer. To examine if the membership to the NHIS contributes to increasing access and utilization of health services, the following maternal, newborn and child health (MNCH) service access and utilisation measures were selected: 1) ANC 4+, defined as the percentage of at least four antenatal care visits during pregnancy among women who had a live birth during the two years preceding the survey; 2) Content of antenatal care, defined as the percentage of comprehensive ANC (i.e. blood pressure measured, urine sample taken, and blood sample taken as part of antenatal care) among women who had a live birth during the two years preceding the survey; and 3) Post-natal health checks for newborns within 2 days of delivery, defined as percentage of newborns born in the last two years who received health checks and post-natal care (PNC) visits from any health provider within 2 days of delivery. All women who gave birth in the two years preceding the survey were included in the analysis. Pearson design-based F test was used to explore the association of NHIS membership and background characteristics of women aged 15–49 years dully interviewed.

A generalised linear model (GLM) with binomial-family logit-link was used to estimate the effect of NHIS membership on each of the service utilisation indicators, adjusting for relevant confounding factors. Socioeconomic status measured by household wealth quintiles, using an asset index, was considered *a priori* as potential confounder for the NHIS membership-service utilisation relationship and so was adjusted for in all the analyses. Other maternal and household characteristics such as mother’s level of education and area of residence were explored for potential confounding. An adjusted Wald test was used to calculate the P-value as a measure of random error in the adjusted regression model.

To examine if NHIS membership can contribute to improved health outcome for children, the study also considered under-five mortality, defined as the probability of dying before the fifth birthday. Every child recorded in the complete birth history dataset who was born within five years preceding the survey was included. The death before the child’s fifth birthday or the woman’s date of interview was estimated using the Kaplan-Meier failure method. The hazard function was estimated for key maternal and household characteristics as well as wealth quintiles. The Cox proportional hazard regression model was used to estimate the effect of NHIS membership on the incidence of under-five deaths adjusted for wealth quintiles and other potential confounders. The birth history data is suitable because it records the three key variables of survival data, i.e. date of birth of child, date of death of child (or age at death), and event status - death or alive.

All analyses were adjusted for survey design characteristics (i.e. sampling weight, cluster sampling, and stratification). The analyses were performed using Stata version 13 (StatCorp, College Station, Texas, USA).

## Results

A total of 10,670 women aged 15–49 years were duly interviewed. Of which, 7,310 (69 %) have ever registered with NHIS. Of the number who has ever registered with NHIS, 40 % had valid card as at the day of interview, which was seen by the interviewer. About 40 % of women who have ever registered with the NHIS had a valid insurance card, which was seen by the interviewer (Table 1). There were wide regional variations in national health insurance coverage. For example, only 24 % of women who have ever registered with the NHIS had valid NHIS card in Central region compared with 55 % in Upper West (*P* < 0.0001). Women in the rural areas were more likely to hold a valid NHIS card (42 %) compared with those in urban areas (39 %) (*P* = 0.07). The results also showed that the poorest 20 % were least covered (38 %) by the national health insurance compared with the richest quintile (44 %), although the observed difference is likely to be due to chance (*P* = 0.19). There was strong evidence that recent mothers were more likely to have a valid NHIS card compared to those who did not give birth in the last two last before the survey (F = 15.23; *P* < 0.001) (Table 1).

**Table 1:** Membership of NHIS among women aged 15–49 years by background characteristics, Multiple Indicator Cluster Survey 2011, Ghana

Characteristics	Number of women who have ever registered (%)	Percent of ever registered with valid NHIS card	Pearson design-based F test
Region			*P* < 0.0001
Western	638 (62.4)	41.4	
Central	606 (58.0)	24.2	
Greater	1163 (56.1)	27.5	
Volta	567 (69.0)	39.3	
Eastern	917 (74.1)	41.5	
Asante	1535 (77.4)	52.7	
Brong Ahafo	839 (83.6)	43.0	
Northern	514 (68.2)	34.9	
Upper East	305 (75.4)	44.7	
Upper West	226 (80.1)	54.7	
Area of Residence			*P* = 0.07
Urban	4091 (70.9)	38.6	
Rural	3219 (66.3)	42.4	
Age (Years)			*P* = 0.01
15–19	1279 (67.4)	34.7	
20–24	1138 (68.0)	39.8	
25–29	1209 (68.4)	40.0	
30–34	1173 (71.6)	45.3	
35–39	1019 (71.2)	41.5	
40–44	823 (68.9)	39.1	
45–49	669 (65.4)	43.3	
Had births in last two years			*P* < 0.001
No	5461 (67.4)	38.6	
Yes	1849 (73.1)	45.5	
Education			*P* = 0.07
None	1356 (61.0)	40.4	
Primary	1248 (61.6)	39.2	
Middle/JHS^1^	3111 (71.9)	38.4	
Secondary+	1595 (77.8)	44.8	
Wealth Index Quintile			*P* = 0.19
Poorest	715 (43.1)	38.0	
Second	1183 (63.0)	40.7	
Middle	1442 (68.6)	37.3	
Fourth	1768 (75.4)	40.3	
Richest	1973 (74.6)	43.5	
Total	7310 (68.8)	40.3	

^1^JHS represents Junior High School

### Association between NHIS membership and MNCH Service utilisation

Antenatal care visits during pregnancy are a key intervention for linking women to skilled delivery and immediate postnatal care. The results showed 87 % of at least four antenatal care visits among women who had live births during the two years preceding the survey. Women with valid NHIS card confirmed by the interviewer were more likely to make the minimum recommended visits (Prevalence = 92.3 %; 95 % CI = [89.2, 94.5]) than those without valid card or no card at all (Prevalence = 83.9 %; 95 % CI = [81.0, 86.4]). After adjusting for the confounding effect of household wealth quintiles, the odds of making at least four ANC visits among women with valid NHIS card were about two times among those without valid card or no card at all (adjusted OR = 1.94; 95 % CI = [1.28, 2.95]; *P* < 0.01) (Table 2).

**Table 2:** Adjusted effect of NHIS membership on Maternal Newborn and Child Health (MNCH) service utilisations among women who had live births during the two years preceding the survey, Multiple Indicator Cluster Survey 2011, Ghana

MNCH Services	Odds Ratio adjusted for Rural_Urban, Education and Wealth Quintiles		Adjusted Wald *P*-value
	Adjusted OR	95 % CI	
ANC4+	1.94^1^	[1.28,2.95]	*P* < 0.01
Comprehensive ANC	2.05^2^	[1.46,2.90]	*P* < 0.0001
PNC for newborns within 48 h	1.08^1^	[0.80, 1.45]	*P* = 0.62

^1^Adjusted for Wealth Quintiles

^2^Adjusted for Rural_Urban, Mother’s Educational level, and Wealth Quintiles

Among those women who have given birth to a child during the two years preceding the survey, 89 % had their blood pressure measured, and their urine specimen and blood sample taken. Coverage for these antenatal care services was common among women with valid NHIS card (Prevalence = 94 %; 95 % CI = [92.2, 95.5]) compared with those without valid card (Prevalence = 86 %; 95 % CI = [84.1, 88.2]). After adjusting for the confounding effects of household wealth quintiles, area of residence, and education, there was strong evidence that women with valid NHIS card were about two times more likely to receive comprehensive ANC than those without valid card or no card at all (adjusted OR = 2.05; 95 % CI = (1.46,2.90); *P* < 0.0001) (Table 2).

With regards to PNC visits, only 15 % of newborns received their first Post-natal care visit within 48 h of delivery. The proportion was similar between NHIS cardholders (16 %) and non-NHIS cardholders (15 %). After accounting for the confounding effect of household wealth, there was no evidence of association between NHIS membership and odds of PNC visits for newborns within 48 h following delivery (adjusted OR = 1.08; 95 % CI = (0.80, 1.45); *P* = 0.62) (Table 2).

### Association between NHIS membership and under-five mortality

**Fig. 1:**
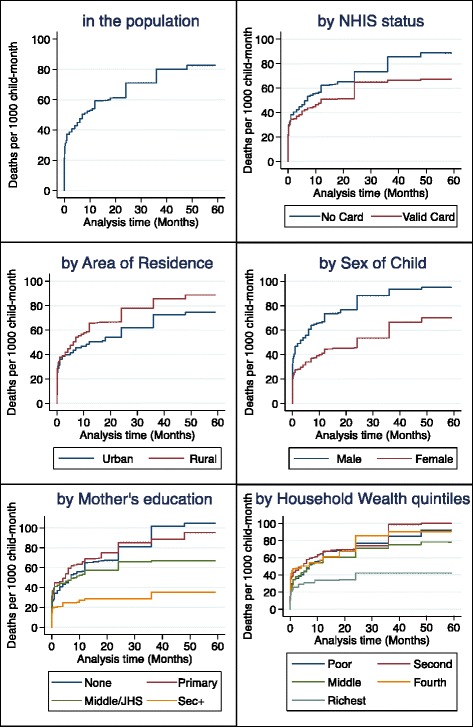
Kaplan Meier failure estimates (mortality per 1000 child-month) in the 5 years preceding the Multiple Indicator Cluster Survey 2011 among children under-five, Ghana

The incidence of under-five deaths in the five years preceding the survey was about 82 deaths per 1,000 child-months by the date of interview of woman (Fig. 1). The deaths appeared to be more concentrated during the first year of life as indicated in the many steps shown in the Kaplan-Meier graphs (Fig. 1). Each step in the graph indicates at least one death; the closer the steps the more frequent the death was occurring. The frequency with which children under-five died was only slightly slower among those whose parents had a valid NHIS card (hazard rate = 67 deaths per 1,000 child-months) compared with those whose parents did not (hazard rate = 89 deaths per 1,000 child-months). After adjusting for the confounding effects of household wealth, sex of child, and mother’s education, the data provides no evidence of association between NHIS membership and incidence of under-five deaths (adjusted hazard rate = 0.86; 95 % CI = [0.64, 1.14]; *P* = 0.30) (Table 3).

**Table 3:** Cox proportional hazard model of the adjusted effect of NHIS membership on incidence of deaths among children under-five during the five years preceding the survey, Multiple Indicator Cluster Survey 2011, Ghana

Characteristics	Crude hazard rate [95 % CI]	Wald *P*-value	Adjusted hazard rate [95 % CI]	Adjusted Wald *P*-value
NHIS membership		*P* = 0.15		*P* = 0.30
No valid card/Valid card not seen	1		1	
Have valid card seen	0.81 [0.60, 1.08]		0.86 [0.64, 1.14]	
Area of Residence		*P* = 0.17		
Urban	1			
Rural	1.22 [0.92, 1.61]			
Mother’s Education		*P* = 0.01		*P* = 0.12
None	1		1	
Primary	0.99 [0.71, 1.37]		0.98 [0.69, 1.39]	
Middle/JHS	0.76 [0.55, 1.05]		0.78 [0.55, 1.10]	
Secondary+	0.37 [0.20, 0.69]		0.46 [0.21, 0.99]	
Wealth Index Quintile	*P* = 0.11		*P* = 0.56	
Poorest	1		1	
Second	1.09 [0.78, 1.51]		1.17 [0.83, 1.63]	
Middle	0.90 [0.63, 1.28]		1.01 [0.70, 1.46]	
Fourth	1.05 [0.70, 1.57]		1.24 [0.80, 1.92]	
Richest	1.52 [0.30, 0.89]		0.77 [0.40, 1.48]	
Sex of Child		*P* < 0.01		*P* < 0.01
Male	1		1	
Female	0.64 [0.50, 0.84]		0.65 [0.51, 0.85]	
Mother’s age at birth (Years)		*P* = 0.29		
Less than 20	1			
20–34	0.76 [0.52, 1.12]			
35–49	0.91 [0.61, 1.36]			

## Discussion

Ghana’s national health insurance scheme was implemented as a demand side intervention to remove financial barriers to promote access and utilization of health services. This study seeks to investigate the association of national health insurance membership with key maternal and child health services utilisation and under-five mortality.

The results support the role of NHIS membership in improving access and utilization of maternal and child health services including, antenatal care and content of antenatal care. These results are consistent with Owoo et al. [8], Arthur [22], and Brugiavini and Pace [10]. The results also suggest that women in the richest wealth categories utilize maternal health services more intensively compared to women in the poorest wealth category. Similar to findings by Arthur [22], household wealth plays a significant role in maternal health care utilisation, even after controlling for health insurance. This suggests that there are other costs related to maternal and newborn health care, which are borne by clients or are not catered for under the health insurance scheme. Clients still pay for some medicines and laboratory tests covered under the NHIS because the service providers evaluate the actual cost to be higher than the NHIS flat fee. For example, laboratories charge for blood cultures because they cost the service provider more than the NHIS can reimburse. There is also resistance to the common antibiotics covered under the NHIS, and so treatment of infections requires more expensive antibiotics. Clients pay for these prescriptions at the pharmacies. They also pay for antihypertensive dugs for women with high blood pressure. The cost of delivery kits, and diagnostic imaging services such as CT scans and MRI, are often born by the clients.

With regard to newborns, there was no evidence that NHIS membership plays a critical role in post-natal check-up for newborns within 48 h of delivery. A possible reason is that babies who were delivered in the clinic were more likely to undergo health checks within 48 h of delivery, especially when the mother spends more than two days in the clinic after delivery. Once the newborns are presented at the clinic for post-natal check-up, they are provided with the requisite services irrespective of the NHIS membership status of the mother. The problem rests with deliveries at home or with Traditional Birth Attendants; babies born under these circumstances have limited access to newborn services, possibly because their mothers do not appreciate the benefits of health checks for their newborns or they live in remote communities with limited transport services or sometimes they are ignorant about newborn illness. Sociocultural factors also play a role because in some cultures babies are not out-doored until after naming ceremony is performed within seven days of delivery.

On child health outcomes, the study failed to show evidence of association of NHIS membership with under-five mortality. These results were consistent with Ansah et al. [19] who found that, although free health care increases health seeking behaviour, it does not lead to a positive impact on child health outcomes. The apparent lack of impact of NHIS on child health outcomes, for which the study had good statistical power, challenges the assumption that increased access as a result of free health care translate into health benefits. However, there remain equity arguments for providing free healthcare and there may be unmeasured effects of removing out of pocket payments on children’s health. A number of reasons may explain this lack of effect. It may be that increases in health care utilization among those with valid NHIS card compared to those without valid card were too modest to produce a clear effect on health. It is also possible that user fees may not be the major financial barrier to access to healthcare in the formal health sector in Ghana, so removing them may have had relatively limited impact. Indirect costs, including opportunity costs (e.g. the cost of time spent away from work), may be more important and are not as easily modifiable as direct costs of care [23, 24]. If indirect costs limit some patients from accessing free care or eats deep into household disposal income, this is likely to have had its greatest effect on the poorest who are expected to benefit from effective healthcare. Sicuri et al. [25] have shown in studies conducted in Kenya, Tanzania and Ghana that indirect costs constitute a large proportion of household and health system costs for malaria treatment, which has implication for health outcomes, as malaria has been associated with the burden of under-five mortality. Non-financial barriers such as distance from health care facilities, negative perceptions of quality of health care services, and when to use these services, may also be important in determining health-seeking behaviour and will not be affected by removing user fees.

The authors recognised some methodological challenges in respect of the apparent lack of association between NHIS membership and under-five mortality. For example, estimation of NHIS membership is immediate, i.e. an assessment of possession of valid card the day the woman was interviewed. This presents methodological challenges when comparing to estimates that are not temporally aligned - a woman could present a valid NHIS membership the day of survey interview despite not having it when her baby died few years before. The child death would be registered as a death occurring for a valid NHIS member. For some, NHIS membership could be a consequence of previous catastrophic expenditure related to child death.

Nonetheless, these findings are particularly important for policymakers, given the size of investments in providing free health care that could be shifted from other priorities on the basis of the assumption that increased access as a result of free health care translate into health benefits. The findings highlight the need to look more into developmental issues generally that can have direct impact on health and wellbeing. There are trends that must not be ignored - the nature of health problems is changing in ways that were only partially anticipated, and at a rate that was wholly unexpected. The effects of ill-managed urbanization accelerate nationwide transmission of communicable diseases. Today, dehydration caused by severe diarrhoea is a major cause of morbidity and mortality among young children in Ghana, although the condition can be easily treated with oral rehydration therapy (ORT). Exposure to diarrhoea-causing agents is frequently related to the use of contaminated water and to unhygienic practices in food preparation and disposal of excreta. These are largely developmental problems that need to be tackled head-on.

## Conclusions

National health insurance membership is associated with increased health care utilisation as measured by antenatal care visits but not with under-five mortality.
